# Erythropoietin receptor in B cells plays a role in bone remodeling in mice

**DOI:** 10.7150/thno.45845

**Published:** 2020-07-09

**Authors:** Naamit Deshet-Unger, Albert Kolomansky, Nathalie Ben-Califa, Sahar Hiram-Bab, Dafna Gilboa, Tamar Liron, Maria Ibrahim, Zamzam Awida, Anton Gorodov, Howard S. Oster, Moshe Mittelman, Martina Rauner, Ben Wielockx, Yankel Gabet, Drorit Neumann

**Affiliations:** 1 Department of Cell and Developmental Biology, Sackler Faculty of Medicine, Tel Aviv University, Israel.; 2 Department of Anatomy and Anthropology, Sackler Faculty of Medicine, Tel Aviv University, Israel.; 3 Department of Medicine A, Tel Aviv Sourasky Medical Center, Sackler Faculty of Medicine, Tel Aviv University, Israel.; 4 Department of Medicine III, Dresden University Medical Center, Germany.; 5 Institute for Clinical Chemistry and Laboratory Medicine, Technische Universität Dresden, Dresden, Germany.

**Keywords:** bone marrow, Pro-B cells, lymphocytes, osteoclastogenesis, transdifferentiation, erythropoietin, cFMS/CD115/CSF1R

## Abstract

Erythropoietin (EPO) is a key regulator of erythropoiesis. However, EPO receptors (EPO-Rs) are also expressed on non-erythroid cell types, including myeloid and bone cells. Immune cells also participate in bone homeostasis. B cells produce receptor activator of nuclear factor kappa-Β ligand (RANKL) and osteoprotegerin (OPG), two pivotal regulators of bone metabolism. Here we explored the ability of B cells to transdifferentiate into functional osteoclasts and examined the role of EPO in this process in a murine model.

**Methods:** We have combined specifically-designed experimental mouse models and *in vitro* based osteoclastogenesis assays, as well as PCR analysis of gene expression.

**Results:** (i) EPO treatment *in vivo* increased RANKL expression in bone marrow (BM) B cells, suggesting a paracrine effect on osteoclastogenesis; (ii) B cell-derived osteoclastogenesis occured *in vivo* and* in vitro,* as demonstrated by B cell lineage tracing in murine models; (iii) B-cell-derived osteoclastogenesis *in vitro* was restricted to Pro-B cells expressing CD115/CSF1-R and is enhanced by EPO; (iv) EPO treatment increased the number of B-cell-derived preosteoclasts (β3^+^CD115^+^), suggesting a physiological rationale for B cell derived osteoclastogenesis; (v) finally, mice with conditional EPO-R knockdown in the B cell lineage (cKD) displayed a higher cortical and trabecular bone mass. Moreover, cKD displayed attenuated EPO-driven trabecular bone loss, an effect that was observed despite the fact that cKD mice attained higher hemoglobin levels following EPO treatment.

**Conclusions:** Our work highlights B cells as an important extra-erythropoietic target of EPO-EPO-R signaling and suggests their involvement in the regulation of bone homeostasis and possibly in EPO-stimulated erythropoietic response. Importantly, we present here for the first time, histological evidence for B cell-derived osteoclastogenesis *in vivo*.

## Introduction

Erythropoietin (EPO), the principal hormone regulating red blood cell production, acts by binding to the EPO receptor (EPO-R) on erythroid progenitors, thereby promoting their survival, proliferation, and differentiation 1. Recombinant human EPO (rHuEPO, hereafter EPO) is widely prescribed for treating anemia in patients with advanced chronic kidney disease 2, as well as anemia associated with certain hematological disorders, primarily myelodysplastic syndromes and multiple myeloma 3, 4. It has now become clear that EPO has additional non-erythroid effects (e.g. 5-8), including an EPO-associated decrease in bone mass in humans 9. Similarly, high EPO levels in mice were found to be associated with a substantial decrease in bone mass 8, 10-13. An important observation that is central to the current study, is the evidence provided by us 10, 14 and others 15, 16 for the existence of functional EPO-R signaling in myeloid cells. Both preosteoclasts 10 and osteoblasts 11, 17-19 express EPO-R and respond to EPO. We have previously reported that the enhanced bone resorption induced by EPO treatment involves stimulation of osteoclastogenesis combined with inhibition of osteoblast mineralization and bone formation 10, 11.

The contribution of other bone marrow (BM) cells, specifically B cells, to EPO driven bone loss has not yet been resolved.

Bone turnover is regulated by the coordinated actions of the canonical monocyte-derived multinucleated osteoclasts, which mediate bone resorption, and mesenchymal stroma cell derived osteoblasts, which mediate bone formation 20. Monocyte differentiation to osteoclasts is dependent on macrophage colony stimulating factor **)**M-CSF**(**, its cognate receptor CSF1-R (also cFms, or CD115) 21 and receptor activator for nuclear factor kappa B (RANK) ligand RANKL 22. Although both RANKL and its decoy receptor osteoprotegerin (OPG) 22, 23 are mainly produced by osteoblasts and osteocytes 24, B cells were also shown to produce these molecules 25, 26, thus placing these cells as important regulators of bone homeostasis *via* paracrine signals 27.

Osteoclasts and B cells arise from distinct myeloid and lymphoid progenitors, respectively 28, and follow distinct differentiation pathways. In the bone marrow (BM), B cell maturation progresses from the pro-B cell stage through pre-B and immature B cell stages 29. However, previous studies have revealed that “change of fate” among early B cell precursors can occur. In line with the current paper, several reports demonstrated that early BM B cells are capable of differentiation into macrophages 29-32, the well-established osteoclast precursors. The occurrence of non-canonical osteoclastogenesis from B cells has been suggested but is still controversial 33-36. Indeed, some concern accompanied previous reports since the presence of residual monocytic cells in isolated B cell culture could not be entirely ruled out 37, and evidence for the *in vivo* occurrence of this pathway is lacking.

Here we present data suggesting that EPO treatment induces bone loss at least partly through its effect on B cells, both by increasing the expression of osteoclastogenic molecules (e.g. RANKL) on these cells as well as by enhancing the ability of the B cells to transdifferentiate into functional osteoclasts. In this respect, utilizing a lineage tracing approach, we were able to demonstrate the occurrence of osteoclasts originating from BM B cells *in vivo*. Our conclusions are further strengthened by the observation that a conditional knockdown of the EPO-R in B cells results in a higher bone mass phenotype.

## Methods

### Experimental animals and administration of rHuEPO

Mouse handling and all experimental procedures were approved by the Institutional Animal Care and Use Committee of the Tel-Aviv University (permit numbers: M-14-043, 01-016-010) and were performed in accordance with the approved guidelines.

Experiments were conducted on 8 to 16-week-old female C57BL/6J wild-type mice (Envigo, Israel), and 11-week old female MB1-Cre; EPO-R^fl/fl^ (cKD) mice and their corresponding controls. The use of female mice is in accordance with our previous studies on EPO-R in bone and in the immune system which were all conducted in female mice, e.g. 10, 38, 39. MB1-Cre; R26R-EYFP and CD19-Cre; R26R-EYFP transgenic mice were used for the lineage tracing experiments. All transgenic mice were of the C57BL/6J genetic background. MB1-Cre^+/-^ mice were kindly provided by Prof. M. Reth, Max Planck Institute of Immunobiology, Freiburg, Germany. Animals were fed ad libitum (1318 Forti, Altromin International, Germany) and kept in 21±1°C ambient temperature in cages (up to 5 mice per cage) with 12-hour light/darkness cycle. All transgenic mice were kept in specific pathogen free (SPF) facility. rHuEPO (EPO) (Epoetin α, Eprex®, Janssen) at a dose of 180 U was administered subcutaneously (s.c.), three times a week for one or two weeks as indicated. In the experiments involving EPO administration to transgenic animals, littermates of the same genotype were allocated in a 1:1 manner into EPO and diluent treatment groups (diluent = sterile saline 0.9%). Based on the literature and on our own experience, a minimum of 7 mice per group is required to attain satisfactory statistical power in *in vivo* studies. Because we investigated the contribution of B cells' EPO-R in the overall skeletal effects of EPO, we elected a sample size of 10±1 mice.

### Flow cytometry and sorting of B cells

BM cells were flushed from femurs, tibias, and the pelvic bone and red blood cells were lysed using ACK lysis buffer (Quality Biological, Gaithersburg, MD). The cells were then stained for 30 min at 4°C with conjugated anti-mouse antibodies: B220 - FITC/PE, CD19 - PE/FITC/efluor450, IgM - PerCP-efluor710/APC, CD43 - PE-Cy7, CD115 (cFms, CSF1-R, MCSF-R) - PE/APC, β3 integrin - AlexaFluor-647 and RANKL - PE (eBiosciences and Biolegend, San Diego, CA). After this time cells were washed with PBS containing 2% FBS and either sorted on a BD FACS Aria II (BD Biosciences, San Jose, CA) or analyzed by Gallios flow cytometer and Kaluza software (Beckman Coulter, Indianapolis, USA).

### Osteoclast differentiation *in vitro*

Isolated cells were seeded on tissue culture-treated 96-well plates in α-MEM containing 10% FBS, M-CSF (2% of culture supernatants from CMG 14-12 cells, containing 1.3 µg/ml M-CSF (CMG medium) 10, 40), and 50 ng/ml recombinant murine RANKL (R&D Systems, Minneapolis, MN). Culture medium was replaced every 2-3 days. After 5-8 days, the cells were stained for tartrate-resistant acid phosphatase (TRAP) (Sigma-Aldrich, MO, USA). TRAP^+^ cells with ≥ 3 nuclei were counted as mature osteoclasts. Osteoclast surface area (OC area) was measured using ImageJ software (NIH, Bethesda, MD).

### Pit resorption

Isolated BM B cells from C57BL/6J mice were cultured in 96-well Corning^©^ Osteoassay surface multiwell plates (Sigma-Aldrich, MO, USA) in α-MEM containing 10% FBS, 100 ng/ml RANKL, and 2% culture supernatants from CMG 14-12 cells as a source of M-CSF. Culture medium was replaced every 2-3 days. After 8-10 days, the media were removed and the cells were bleached with 5% sodium hypochlorite (NaClO) for 5 min. The wells were washed with distilled water and allowed to dry overnight. The results (generation of the pits in the bone surface) were viewed using light microscopy. Resorption area was measured using ImageJ software (NIH, Bethesda, MD).

### Real-time quantitative PCR

Total RNA was extracted from sorted B220^+^CD19^+^ cells (total B cells), B220^-^CD19^-^ (non-B BM cells) and B220^+^CD19^+^IgM^-^CD43^high^ (Pro-B cells), with the Quick-RNA microprep kit (Zymo research, California, USA) according to the manufacturer's instructions. To extract RNA from whole bone (including BM) tibial heads were first mechanically homogenized and then subjected to a standard column RNA extraction procedure using TriRNA Pure kit (Cat.# TRPD200, Geneaid, New Taipei city, Taiwan). cDNA was synthesized using the qScript cDNA synthesis kit (Quantabio, Massachusetts, USA). “Real-time” quantitative PCR (RT-qPCR) was performed on StepOnePlus instrument using SYBR Green reagent (both from Applied Biosystems, California, USA). In some experiments, gene expression was assessed by the TaqMan method (EPO-R: Mm00833882; Hprt: Mm00446968). Relative gene expression was calculated using the ∆∆ct method following normalization to the expression of HPRT as a house keeping gene. All RT-qPCR experiments were performed in triplicates.

### Immunofluorescent staining

In the *in-vitro* experiments, cells were cultured on Vision 96-well plates (4titude, Wotton, UK) in α-MEM containing 10% FBS, 2% CMG medium, and 50 ng/ml RANKL. The medium was replaced every 2-3 days. After 5-8 days, cells were fixed in 4% PFA and stained with rabbit polyclonal anti-GFP alexa-Fluor-488-conjugate (Abcam, Cambridge, MA) and DRAQ5^TM^ as a nuclear stain (Thermo Fisher Scientific, Waltham, MA). Images were obtained using STED confocal microscope (LAS-AF, Leica, Germany). Following the acquisition of the florescent images, TRAP staining was used to label osteoclasts and images were collected at the same coordinates as the fluorescence readings.

In order to demonstrate the presence of osteoclasts in bone tissue sections, lumbar vertebrae were fixed in 4% paraformaldehyde and decalcified in 12.5% EDTA for 10-14 days at room temperature on a shaker. The bones were then immersed overnight in 30% sucrose and embedded in O.C.T. compound (Scigen Scientific Gardens, CA, USA) for subsequent sectioning using a cryostat (Leica CM 1950, Leica BIOSYSTEMS-, Germany). Bone sections were stained using chicken anti-GFP followed by goat anti-chicken Alexa Fluor 488 (both from Abcam, Cambridge, UK). After scanning the bone sections by fluorescence microscopy, specimens were subjected to conventional histochemical TRAP staining to avoid inadvertent washout of the antibodies and/or chemical damage to the conjugated fluorophores. DAPI was used as a nuclear stain.

### Micro-Computed tomography (µCT)

Femora (one per mouse) were examined using the µCT50 system (Scanco Medical AG, Switzerland) as reported previously 41, 42. Briefly, scans were performed at a 10-μm resolution. The mineralized tissues were segmented by a global thresholding procedure 43. Trabecular bone parameters were measured in the secondary spongiosa of the distal femoral metaphysis, which was further divided into a proximal and a distal half. Cortical parameters were determined in a 1mm height ring in the mid-diaphyseal region. Volumetric bone mineral density (vBMD) was calculated using the proprietary Scanco software in reference to a calibrated phantom and expressed as mg hydroxyapatite per cm^3^ of tissue (mgHA/cm^3^).

### Statistical analysis

Values are expressed as mean ± SEM (standard error of the mean). When comparing two groups of variables, either the parametric unpaired Student's *t-*test (when n > 5) or the nonparametric Mann-Whitney test (e.g. when n ≤ 5 or unequal variance) were used for calculating statistical significance. In experiments with >2 groups of variables either 1-way or 2-way ANOVA were applied. Post-hoc tests for multiple comparisons were performed when appropriate. Significant difference between groups was defined as *p* < 0.05. Statistical analysis was performed using GraphPad Prism 7 (San Diego CA, USA).

## Results

### EPO treatment *in vivo* induces the expression of surface RANKL on bone marrow B cells and stimulates their transdifferentiation into osteoclasts *in vitro*

**Figure 1:**
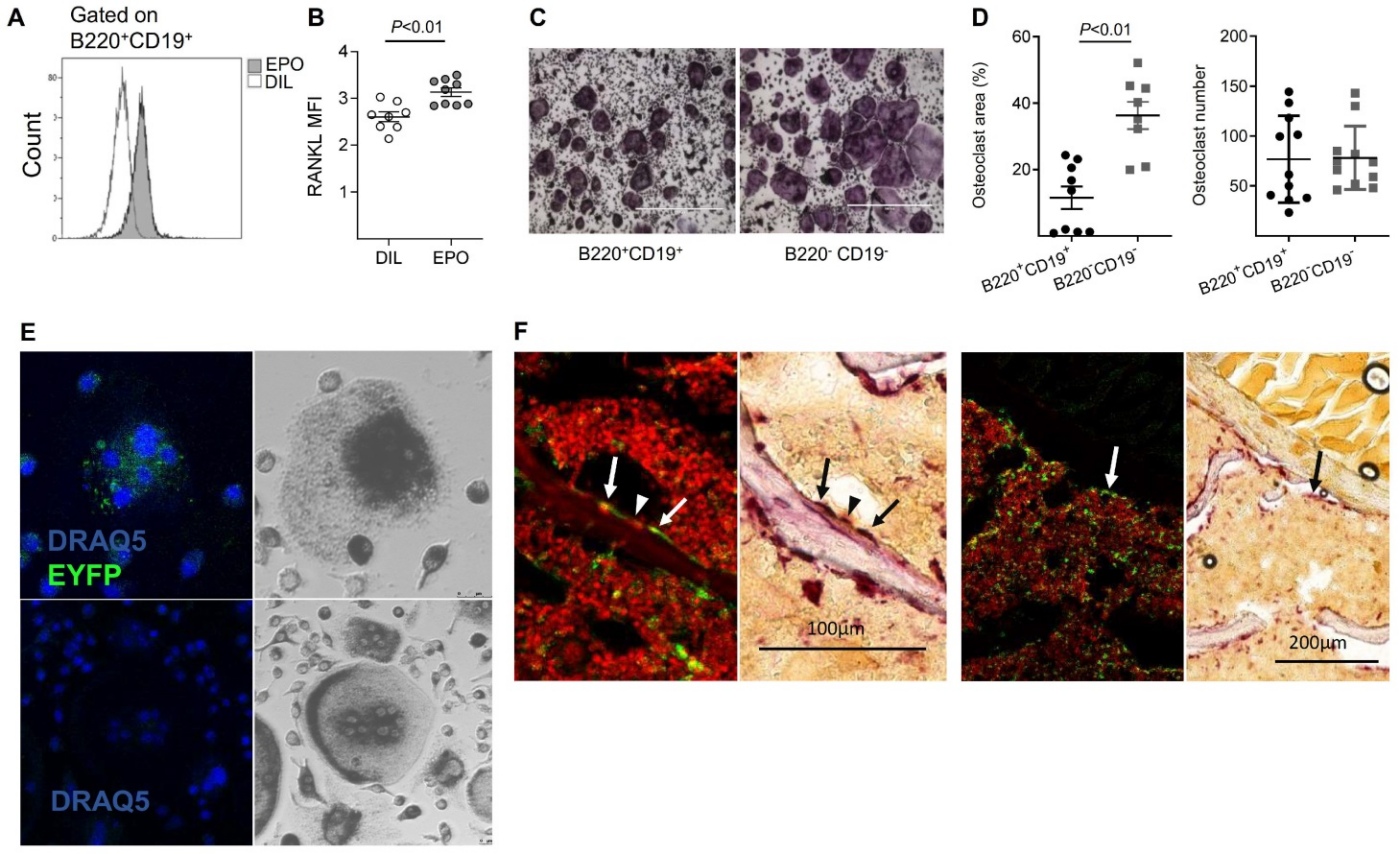
** EPO may regulate bone metabolism *via* bone marrow (BM) B cells by a dual mechanism, involving both an effect on the expression of RANKL by these cells and B cell-derived osteoclastogenesis. (A)** Representative histogram of flow cytometry analysis of RANKL-expressing BM B cells (B220^+^CD19^+^). **(B)** Surface expression of RANKL by BM B cells in EPO- versus diluent-treated female mice as measured by mean fluorescence intensity (MFI). Data are mean ± SEM, n = 8-9 mice in each group. **(C)** TRAP staining (indicating differentiated osteoclasts) of sorted B220^+^CD19^+^ (left) and B220^-^CD19^-^ (right) cells (180,000 cells per well) cultured with M-CSF and RANKL. Representative images were acquired at ✕4 magnification. **(D)** Total area (left) and number (right) of TRAP-positive multinucleated (≥ 3 nuclei) cells. Data are mean ± SEM, n = 8-9 mice in each group. **(E)** B-cell-derived osteoclastogenesis *in vitro* as demonstrated by the differentiation of sorted B220^+^CD19^+^ BM cells derived from CD19-Cre;R26R-EYFP B cell-specific reporter mice (upper panels) and R26R-EYFP (Cre negative) mice (lower panels), which served as a negative control for the anti-GFP staining. Blue - nuclear stain (by DRAQ5©), green - EYFP (enhanced by Alexa Fluor 488-conjugated anti-GFP). Right - conventional TRAP staining performed on the following day (dark gray) (✕20 magnification). **(F)** Lineage tracing *in vivo*: B-cell-derived osteoclastogenesis as demonstrated by immunofluorescent staining of formalin fixed OCT-embedded (FFOE) bone sections derived from MB1-Cre;R26R-EYFP female mice. The EYFP^+^/TRAP^+^ (the former signifying B cell origin) osteoclasts (arrow) are shown in paired panels where the left side shows the fluorescent staining followed by a conventional TRAP staining (right panels) (both at x20 magnification). YFP^-^ (conventional) osteoclast is marked by an arrowhead. The number of TRAP^+^ osteoclasts's per bone perimeter in the specimen (lumbar vertebra) was 7.2 per millimeter (consistent with previous reports for wild-type C57BL female mice 65, 66.

We 10 and others 12, 13 have previously reported that EPO treatment stimulates bone resorption. Because B cells may contribute to bone resorption *via* the expression of RANKL 25, 44, 45 (reviewed 26, 27, 29), we examined whether EPO administration *in vivo* stimulates B cells to express RANKL. We found that, in normal mice, EPO administration for one week (three injections of 180 U) resulted in significantly higher expression of membrane-bound RANKL by BM B cells (B220^+^CD19^+^, 3.13 ± 0.09 *versus* 2.6 ± 0.1 in EPO- versus diluent (DIL)-treated controls (Figure 1A-B)). Similarly, EPO treatment resulted in a higher number of RANKL expressing B cells (B220^+^CD19^+^, 4% ± 0.45 *versus* 1% ± 0.16 in EPO- versus DIL-injected mice (Figure S1A-B). These data suggest a strong association between the increase in B cell-associated surface RANKL and the bone loss induced by EPO.

In order to assess the ability of EPO to directly affect B cells, we first examined whether these cells express EPO-R. Indeed, RT-qPCR analysis demonstrated that the total BM B cell population (B220^+^CD19^+^) expresses EPO-R; here, 5T33 multiple myeloma cells served as a negative control for EPO-R expression 38. Moreover, treatment with EPO for one week increased the levels of EPO-R transcripts in total BM (TBM) as well as in isolated BM CD19^+^B220^+^B cells (TBC) demonstrating the functional response of EPO-R in these cells (Figure S1C).

Based on previous reports, in addition to the increased expression of osteoclastogenic factors, we analyzed the ability of BM B cells to transdifferentiate into bone-resorbing osteoclasts in our experimental system. We therefore subjected isolated BM-derived B220^+^CD19^+^ cells as well as B220^-^CD19^-^ cells (containing monocytes - the classical osteoclast precursors) to an osteoclastogenic assay. Following the standard protocol, which included RANKL and MCSF, we clearly observed TRAP^+^ multinucleated osteoclast-like cells in cultures derived from the B220^+^CD19^+^ B-cells. These cultures generated similar numbers of differentiated osteoclasts, although of smaller size than those obtained from the monocyte-containing B220^-^CD19^-^ cells (11% ± 2.9 and 36% ± 3.3 osteoclast area, respectively) (Figure 1C-D). In order to verify the B cell origin of the TRAP^+^ osteoclasts derived from the B220^+^CD19^+^ cell cultures, we used a lineage tracing strategy where the expression of fluorescent EYFP protein in the B-cell lineage is coupled to the B cell-specific CD19 gene 46, 47. CD19^+^EYFP^+^ cells isolated from the BM of CD19-Cre; R26R-EYFP mice were then cultured under osteoclastogenic conditions. After seven days in culture, we were able to identify EYFP^+^/TRAP^+^ multinucleated giant cells in cultures from cells isolated from CD19-Cre;R26R-EYFP mice (Figure 1E, upper panel), but not from R26R-EYFP (Cre negative) controls (Figure 1E, lower panel). Note the presence of mononuclear green fluorescent cells in the figure, which may also represent B- cell derived osteoclast-like cells, based on their TRAP positivity (upper panels).

### Transdifferentiation of B cells into osteoclasts *in vivo*

Next, we attempted to demonstrate B- cell derived osteoclastogenesis *in vivo*. Assuming that B- cell transdifferentiation involves early rather than late B cell precursors, we preferred the MB1 rather than the CD19-driven Cre in this setting, due to the significantly higher recombination efficiency in the early B cell stages 47. Using fluorescent and light microscopy in a sequential manner 48, we carefully examined bone tissue sections derived from vertebrae of MB1-Cre; R26R-EYFP female mice for EYFP^+^ cells adjacent to the endosteal surfaces of the trabeculae that also displayed a strong TRAP signal. Albeit rare events, we were able to locate such cells with a fluorescent as well as a TRAP signal, as shown in Figure 1F. Although the number of EYFP^+^ osteoclasts was relatively low, their presence confirmed our *in vitro* findings that B cells can transdifferentiate into osteoclasts *in vivo*.

### The B cell subpopulation that transdifferentiates into osteoclasts consists of Pro-B cells expressing CSF1-R

**Figure 2:**
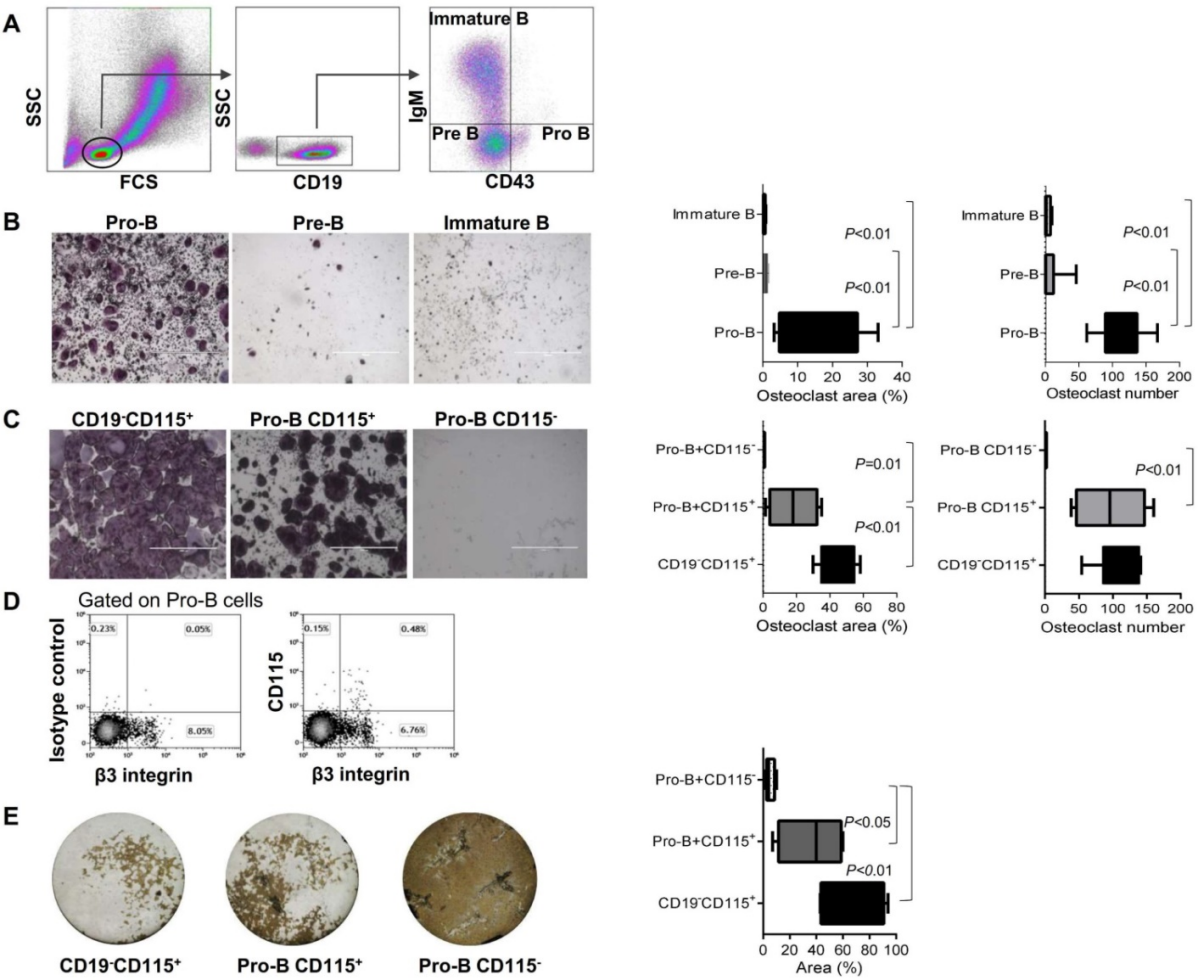
** Lymphoid-osteoclastic differentiation is restricted to CD115^+^ Pro-B cells. (A)** Definition of BM B cell precursor populations by flow cytometry using CD19, surface IgM (IgM) and CD43 antigens. Note, all CD19^+^ cells were also B220^+^ **(B, C)** TRAP staining of osteoclasts derived from the indicated sorted BM cells. Plots represent the percentage of osteoclast area (left) and osteoclast number (right); (B) Left panel - osteoclast differentiation from sorted Pro-B cells (B220^+^CD19^+^CD43^High^IgM), middle - Pre-B cells (B220^+^CD19^+^CD43^Low^IgM^-^) and right - immature B cells (B220^+^CD19^+^CD43^-^IgM^+^)(180,000 cells/well); (C) TRAP staining of osteoclasts derived from the indicated BM sorted cells (10,000 cells per well). Left -positive control of monocyte lineage (CD19^-^CD115^+^). Middle - Pro-B cells expressing CD115. Right - Pro-B cells negative for CD115. n = 5-9 mice in each group; **(D)** Expression of β3 integrin (CD61) by CD115^+^ Pro-B cells **(E)** Pit resorption area from the indicated sorted cells (10,000 cells per well) cultured on calcium phosphate-coated 96-well plates with M-CSF and RANKL. Left - positive control of monocyte lineage (CD19^-^CD115^+^), stopped after 5 days in culture. Middle - Pro-B cells expressing CD115, and right - Pro-B cells not expressing CD115, stopped after 8 days in culture. Note that white area indicates bone resorption while the brown regions are negative for osteoclast activity. Representative images were acquired at x4 magnification. Values in the scatter plot represent the quantification of the pit resorption area (resorbed area is white and non-resorbed is brown). For (B), (C) and (E) the p values were calculated by 1-way ANOVA with Bonferroni post-hoc test. In the “Box and Whisker” plots error bars represent 5-95 percentile range.

We then investigated specifically which subpopulation of B cells can transdifferentiate into osteoclasts. B220^+^CD19^+^CD43^High^IgM^-^ (Pro-B cells), B220^+^CD19^+^CD43^Low^IgM^-^ (Pre-B cells), and B220^+^CD19^+^CD43^-^IgM^+^ cells (immature B cells) were isolated from the total BM cell population of wild-type female mice (see Figure 2A for gating strategy). These B cell subsets (180,000 cells/well) were then subjected to an osteoclastogenic assay, as described in the “materials and methods” section. Pro B cells were the only subset that differentiated into osteoclasts (Figure 2B).

Since CSF1-R (CD115) is essential for osteoclastogenesis 21, we used flow cytometry to isolate a subset of Pro-B cells that express CD115 (B220^+^CD19^+^CD43^High^IgM^-^CD115^+^) and cultured them (10,000 cells/well) with MCSF and RANKL. Remarkably, only CD115^+^ Pro-B cells gave rise to osteoclast-like cells (Figure 2C); here again we confirmed that Pro-B cell cultures generate similar numbers of osteoclasts although of smaller size (Figure 2C). Importantly, neither Pre-B cells nor immature B cells expressing CD115 could differentiate into osteoclasts (Figure 2B), which argues against the possibility that in our cultures residual CD115^+^ cells from the sorting procedure could account for the osteoclasts obtained from the sorted Pro-B cells, either positive or negative for the CD115 marker (data not shown). An additional feature that further supports the preosteoclast phenotype of the CD115^+^ Pro-B cells is the surface expression of β3 integrin (see above) by most of these cells (Figure 2D). Indeed, of the total Pro-B cell population, 0.15% are CD115^ +^/β3^-^ and 0.48% are CD115^+^β3^+^, i.e. 76% of the CD115^+^ express β3. Importantly, the functionality of osteoclasts derived from CD115^+^ Pro-B cells was assessed by means of a pit resorption assay, which indeed indicated that these cells were able to resorb a bone-mimetic surface (Figure 2E). In contrast, cultures of CD115^-^ Pro-B cells did not generate multinucleated osteoclasts and did not display any functional activity (Figure 2C and E). Thus, CD115^+^ Pro-B cells might be considered as B-cell-derived osteoclast precursors.

### EPO induces the expression of CD115 in Pro-B cells and stimulates B cell-derived osteoclastogenesis

**Figure 3:**
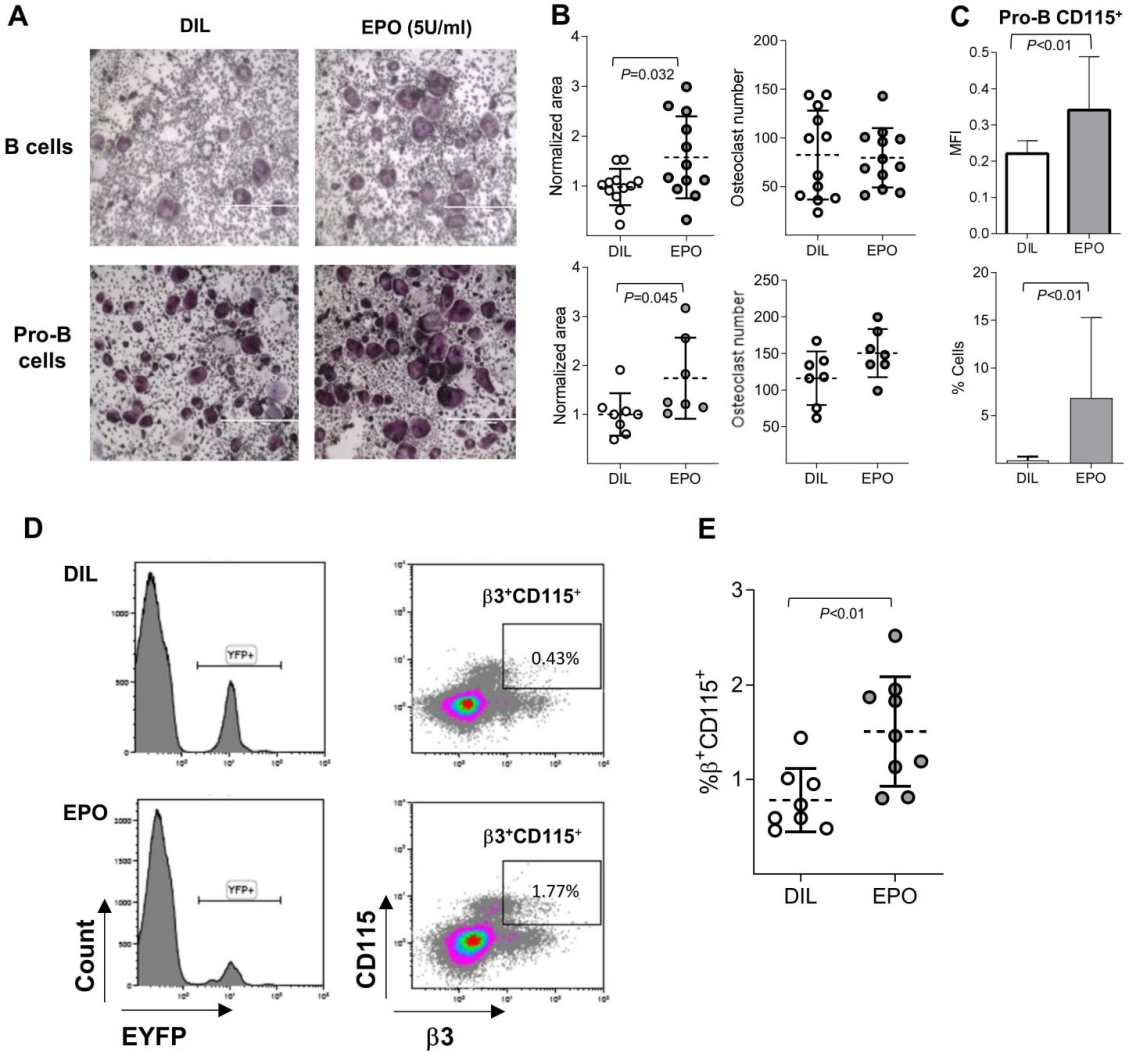
** EPO augments B cell-derived osteoclastogenesis *in vitro* and enriches the pool of B cell lineage-traced osteoclast precursors. (A)** Osteoclast differentiation from sorted BM B cells (B220^+^CD19^+^) and Pro-B (B220^+^CD19^+^CD43^High^IGM^‑^) cells treated with either diluent (DIL) (upper & lower left) or EPO (upper & lower right). Representative images were acquired at x4 magnification. **(B)** Dot plots indicating the osteoclast area (normalized to the mean of the diluent group) and osteoclast number of the corresponding experiments described in (A) (at least 7 mice in each group). **(C)** Flow cytometry analysis of the CD115 expression by Pro-B cells in DIL- versus EPO-injected (for one week) female mice. Upper panel - mean fluorescence intensity, lower panel - % of CD115 cells (n > 14 mice in each group). **(D)** Representative histograms (left panels) and density plots (right panels) of the bone marrow cells isolated from either diluent- (upper panels) or EPO-treated (lower panels) CD19-Cre;R26R-EYFP mice (180 U thrice weekly for 2 weeks). **(E)** Scatter plot indicates the percentage of EYFP^+^/β3^+^/CD115^+^ cells and represent a summary of 8-9 mice in each group. Data are mean ± SEM; p values calculated by Student's t-test.

Having demonstrated above that a subset of B cells can transdifferentiate into osteoclasts and since previous reports also suggested that EPO increases osteoclastogenesis 10-13, we were interested to assess whether EPO stimulates bone loss *via* its effect on B cells. To this end, we cultured BM B cells under osteoclastogenic conditions as already described and subjected them to EPO stimulation. The addition of EPO to sorted BM B cells (CD19^+^/B220^+^) and to Pro-B cells increased the total area of osteoclasts by 57% and 70%, respectively but not their number (Figure 3 A-B). This supports our assumption that EPO increases bone resorption at least in part by stimulating B- cell derived osteoclastogenesis.

Interestingly, the presence of EPO did not affect the osteoclastogenic potential of flow-sorted CD115^+^ Pro-B cells (data not shown), suggesting that the effect of EPO on B cell-derived osteoclastogenesis stems from the upregulation of CD115 (cFms) expression in Pro-B cells, and supporting our previous supposition that these cells serve as B-cell-derived preosteoclasts (Figure 2C). Indeed, the administration of EPO to normal female mice (three injections of 180 U over 1 week 10) resulted in a significant increase (compared to diluent-injected controls) in both CD115 positivity (as measured by flow cytometry) and the proportion of Pro-B cells expressing this receptor (Figure 3C). Of note, EPO (both *in vivo* and *ex vivo*) did not induce osteoclastogenesis from Pre- or immature B cells (data not shown).

### EPO increases the pool of B cell lineage-traced osteoclast precursors

As shown in Figure 3A and B, the addition of EPO to *in vitro* cell cultures enhanced the transdifferentiation of B cells into osteoclast-like cells and *in vivo* EPO administration increased the proportion as well as the MFI values of CD115^+^ Pro-B cells compared to the diluent-injected controls (Figure 3C). We next hypothesized that an enriched pool of B cell-derived osteoclast precursors, induced by EPO treatment, includes not only CD19-expressing (Pro-B) cells but may also include previously committed B cells that have already lost this B cell-defining marker as a part of their transdifferentiation pathway. This premise was tested by treating CD19-Cre;R26R-EYFP mice and their corresponding controls with EPO for two weeks. After this time, we harvested and screened the BM-derived EYFP^+^ cells for surface expression of CSF1-R (CD115) and β3 integrin. Indeed, treatment of CD19-Cre;R26R-EYFP mice with EPO significantly increased the number of EYFP^+^/ CD115^+^/β3^+^ cells in the bone marrow compared to diluent-injected controls (1.5% ± 0.19 *versus* 0.7% ± 0.08, p < 0.05 (Figure 3D-E). The low number of EYFP^+^/CD115^+^/β3^+^ cells found is in agreement with the sparsely distributed B cell-derived osteoclasts observed *in vivo* (Figure 1F). This observation supports our hypothesis that EPO enhances the “B-cell-to-osteoclast” transdifferentiation pathway.

### Knockdown of the EPO-R in B cells is associated with increased bone mass

**Figure 4:**
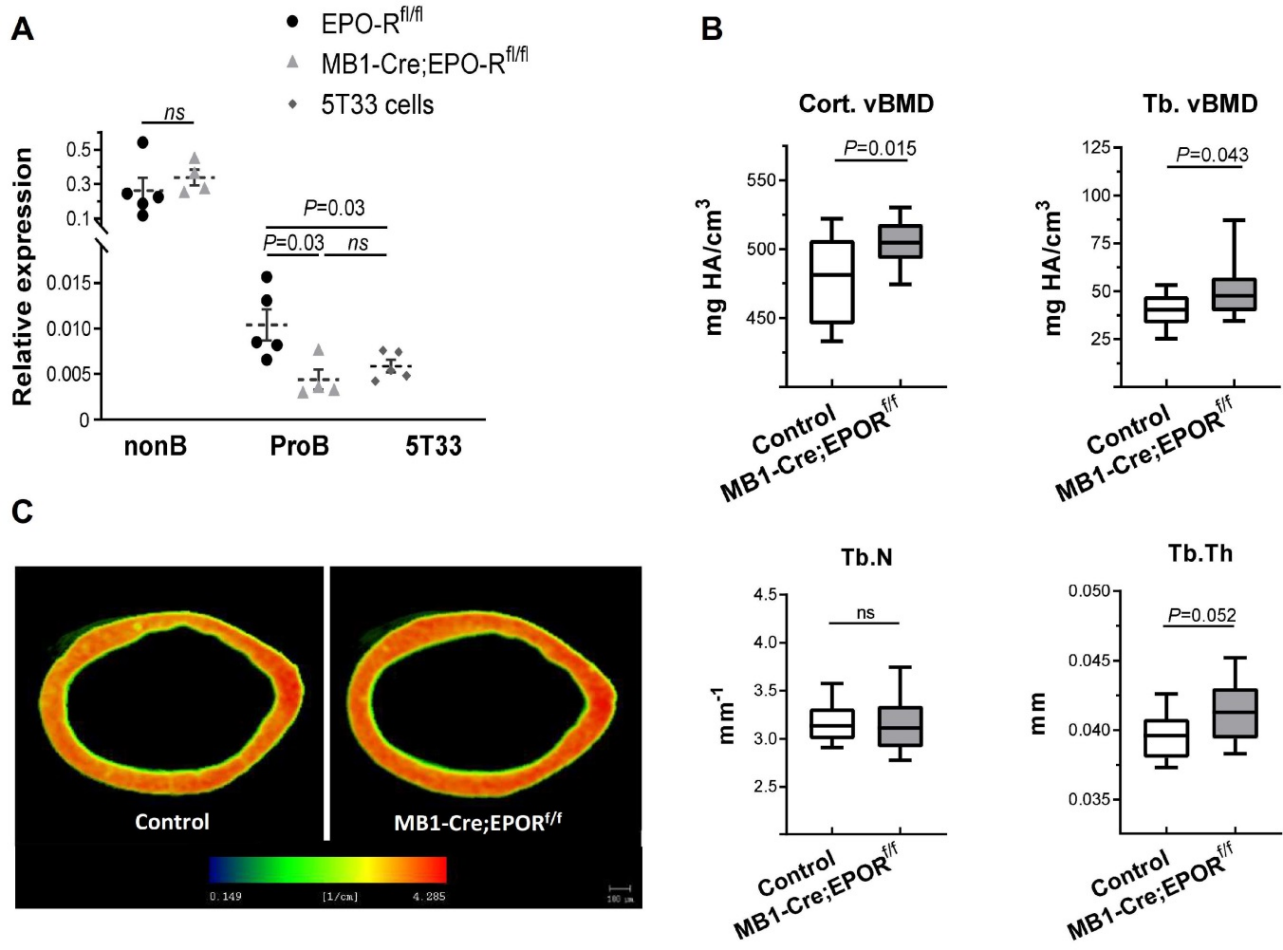
** B-cell-specific EPO-R knockdown is associated with increased bone mass phenotype. (A)** The expression of the murine EPO-R, as measured by RT-qPCR, in the Pro-B cells of the control and MB1-Cre;EPO-R^fl/fl^ mice. The B220^-^/CD19^-^ non-B cell fraction (containing erythroid precursors) and 5T33 myeloma cell line were used as positive and negative controls, respectively 38. Dashed lines and bars are mean ± SEM, n = 4-5 mice in each group. Mann-Whitney nonparametric test was used to calculate the p values shown in the figure. **(B)** Volumetric bone mineral density (vBMD) in the cortical and trabecular bone (upper left and right panels, respectively) as well as trabecular number (Tb.N) and thickness (Tb.Th) (lower left and right panels, respectively) as measured by μCT in the distal femoral metaphyses of transgenic mice carrying a conditional knockdown of EPO-R in the B cells lineage (MB1-Cre;EPO-R^fl/fl^) as compared to MB1-Cre;EPO-R^wt/wt^ controls. n = 11 in each group. **(C)** Representative μCT images of the femoral cortex of the MB1-Cre;EPO-R^fl/fl^ (right) compared to control mice (left). Color spectrum reflects the vBMD gradient across the section of the cortical bone. Data are mean ± SEM, n = 11 in each group. *P* values were calculated by the Mann-Whitney test. In all “Box and Whisker” plots, error bars represent 5-95 percentile range.

To assess the effect of B cell-specific EPO-R signaling on bone mass, we created a murine model of a conditional EPO-R knockdown (cKD) in the B cell lineage, where the Cre-recombinase activity is driven by the MB1 promoter (MB1-Cre;EPO-R^fl/fl^) 47. To validate this model, BM B cell populations were isolated by flow cytometry (based on the expression of CD43 and IgM) and probed for the expression of EPO-R. The results showed that EPO-R was significantly knocked-down already at the stage of Pro-B cells, reaching the background expression levels observed in the 5T33 MM cell line, used as a negative control for EPO-R expression 38 (Figure 4A). As shown in the figure, EPO-R expression in the non-B cells in the BM remained unaffected, nor was there a change in the hemoglobin level in the cKD mice compared to the wild type controls in the absence of EPO injections (data not shown).

The results of µCT analysis of the femurs revealed a higher bone mass in the cKD mice as compared to controls (MB1-Cre;EPO-R^wt/wt^), as measured by both cortical and trabecular volumetric bone mineral density (vBMD, 505 [95%CI 495-516] *vs* 478 [95%CI 458-498] mgHA/cm^3^ and 52.2 [95%CI 42.1-62.3] *vs* 40.8 [34.9-46.7] mgHA/cm^3^, respectively, p < 0.05) (Figure 4B,C). Trabecular thickness (Tb.Th) was slightly higher in the cKD animals (41.3 µm [39.9-42.8] *versus* 39.6 µm [95%CI 38.5-40.7]) though it did not reach statistical significance (p = 0.052). Other µCT parameters are presented in Figure S2.

### B cell-specific EPO-R knockdown attenuates EPO-induced bone loss

**Figure 5:**
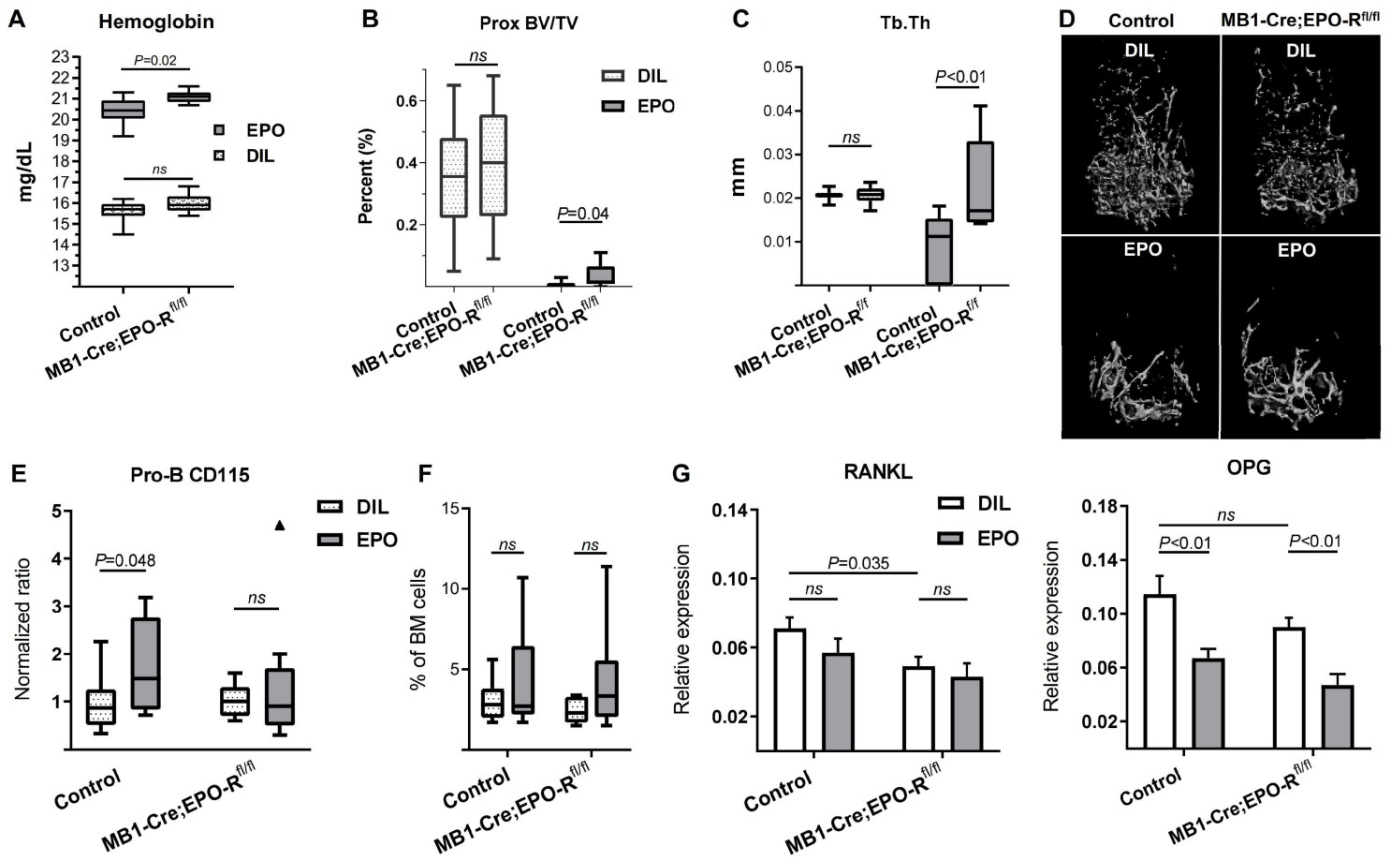
** B-cell-specific EPO-R knockdown attenuates EPO-induced bone loss. (A)** Hemoglobin levels of EPO- versus diluent (DIL)-treated control mice or mice harboring a conditional knockdown of EPO-R in the B cell lineage (MB1-Cre;EPO-R^fl/fl^). p value was calculated by 2-way ANOVA **(B)** trabecular bone volume (BV/TV) and **(C)** trabecular thickness (Tb.Th) in the proximal part of the distal femoral metaphysis of EPO- versus diluent (DIL)-treated control or MB1-Cre;EPO-R^fl/fl^ female mice. **(D)** Representative 3D µCT images of the distal femur of mice described in Figures (B) and (C). **(E)** Proportion of CD115^+^ Pro-B cells (measured by multi-color flow cytometry) in the EPO- versus diluent (DIL)-treated control or MB1-Cre;EPO-R^fl/fl^ mice. Values for each EPO group were normalized to the diluent controls of the same experiment. **(F)** Pro-B cell fraction (out of live cell gate) in the bone marrow of the corresponding experimental animal groups described in (D); **(G)** mRNA expression of RANKL (left) and OPG (right) in the whole bone (proximal tibia) of mice described in (A-F). p values were calculated by 2-way ANOVA. For all panels, n = 9-10 mice in each group. In all “Box and Whisker” plots, error bars represent 5-95 percentile range, except in (E) where error bars are represented by the Tukey method.

To investigate the effect of B cell-specific EPO-R on bone mass during exogenous EPO stimulation (at supraphysiological levels of this hormone), we treated both control and cKD female mice with either EPO or diluent for 2 weeks (180 IU injected 3 times per week), as previously described 10. Interestingly, cKD mice attained higher hemoglobin levels following EPO treatment (21.1 ± 0.09 mg/dL) as compared to the hemoglobin levels measured in the EPO-injected controls (20.4 ± 0.2 mg/dL; p < 0.05, Figure 5A). EPO treatment resulted in a major decrease in the trabecular BV/TV in both the control and cKD mice, reducing the values by 35% and 38.4%, respectively (Figure S3). The decrease in the trabecular vBMD was similar between the control and cKD mice treated with EPO (data not shown). However, the knockdown of B cell-specific EPO-R prevented a virtually complete effacement of the trabecular bone in the proximal part of the distal femoral metaphysis as reflected by a significantly attenuated decline in the proximal BV/TV, probably resulting from a milder thinning of the remaining trabeculae (Figure 5B-D). Moreover, the EPO-induced increase in CD115^+^ Pro-B cells observed in the control mice was absent in the cKD mice (Figure 5E) while the fraction of Pro-B cells out of total BM cells was not altered as a result of EPO treatment (Figure 5F). Taken together, the alterations in the bone mass/microarchitecture in mice with B-cell-specific EPO-R cKD suggest that EPO-R signaling in this lineage plays a role in bone metabolism.

### B-cell-specific EPO-R knockdown affects RANKL/OPG axis under steady-state conditions but not under high dose EPO stimulation

To explore whether the aforementioned alterations in bone microarchitecture in the cKD mice, with or without EPO treatment, may also be related to changes in the RANKL/OPG axis, we analyzed the expression of RANKL and OPG in the whole bone (proximal tibia)^24^ at the end of the treatment period (2 weeks). Despite a significant downregulation of the OPG levels in the EPO-treated mice, cKD was not associated with a differential response of this mediator to pharmacological doses of EPO (ΔΔCT 0.114[95% CI(0.083-0.146)] *vs* 0.067[95% CI(0.051-0.083)] and 0.09[95% CI(0.074-0.106)] *vs* 0.047[95% CI(0.028-0.066)] in the diluent *vs* EPO-treated control and cKD mice, respectively, Figure 5G). RANKL mRNA levels were not significantly affected by EPO administration. However, in line with the µCT data (Figure 4B-C and Figure S3D), cKD mice (without EPO stimulation) exhibited a 31% decrease in the expression of RANKL as compared to their genotypic controls (ΔΔCT 0.071[95% CI(0.056-0.086)] *vs* 0.049[95% CI(0.037-0.075)], respectively, Figure 5G).

## Discussion

The current manuscript highlights the relationship between B cells and bone metabolism and proposes that the effect of EPO on bone metabolism is mediated both through a paracrine pathway and also through an effect on the osteoclastic transdifferentiation potential of early B cells.

We have previously demonstrated that EPO treatment in mice increased the mRNA expression of RANKL in the BM 38. Although the main source of RANKL is osteocytes 49, here we show an EPO-mediated increase in surface RANKL on B cells (Figure 1), which may contribute to EPO- associated bone loss 10. The downregulation in RANKL expression observed in the bones of the MB1-Cre;EPO-R^fl/fl^ (cKD) mice may be a direct or indirect effect of EPO-R deletion in the B cell lineage (Figure 3G). As the mesenchymal cells (including osteocytes), and not the B cells, are the main producers of RANKL 50 and osteoblasts are the main source of OPG 51, we expect that the observed decrease in RANKL mRNA is not primarily due to an alteration in their expression by BM B cells with EPO-R cKD, but rather to an indirect effect of the EPO-R cKD on the main producers of these key molecules. In support of this notion, the levels of RANKL mRNA in BM B cells did not differ between control and cKD animals and OPG transcripts were not detected in any of the experimental animals (data not shown). The question of which bone cells are affected by the lack of EPO-R on B cells, and how this cKD affects RANKL and OPG expression in these non-B cells remains to be addressed.

While the values of BV/TV in the proximal part of the distal femoral metaphysis of the control and cKD mice were similar at steady state (data not shown), EPO treatment of the latter attenuated the decline in this parameter (Figure 5). However, despite experimental evidence of this biological effect, the biomechanical impact of this effect remains questionable since the trabecular density in this region is relatively sparse. It should be noted that the bone effects of EPO-R conditional knockdown, may be underestimated, since the abrogation of EPO-R expression in the Pro-B cells was not complete and reached approximately 60% (Figure 4). On the other hand, substantial impact of B-cell-specific EPO-R knockdown was not anticipated since, apparently, B-cell-driven bone loss is an auxiliary rather than a main mechanism of EPO-induced bone loss. The novelty of these findings lies primarily in the demonstration of a role played by B-cell-specific EPO-R in bone remodeling.

B-cell-specific EPO-R knockdown abrogated the EPO-induced increase in CD115^+^ Pro-B cells without affecting RANKL/OPG levels, as measured in whole bone fragment, in the setting of high dose EPO treatment (Figure 5). In contrast, at physiological levels of EPO (without exogenous EPO administration), cKD an control mice had similar fraction of CD115^+^ Pro-B (data not shown), whereas the former exhibited significantly lower RANKL mRNA levels (Figure 4G), consistent with a bone phenotype. Taken together, these data suggest that under physiological conditions, B-cell-specific EPO-EPO-R signaling affects bone metabolism *via* RANKL/OPG axis, whereas during exposure to high levels of EPO, B-cell-specific EPO-EPO-R contributes to EPO-induced bone loss by an increase in B-cell-derived osteoclastogenesis.

Transdifferentiation of cells is an important and complex process. There have been a number of studies suggesting that B220^+^ cells 33, 34, 36 and B1 peritoneal cells 35 can undergo osteoclastic differentiation although there was also evidence that the contribution of B1 peritoneal lymphocytes to bone loss could be attributed to bacterial or non-specific inflammatory signals 52, 53. Differentiation of B cell subsets into macrophages is a generally accepted process 30, 31, 54, 55. More recently, an early Pro-B cell subpopulation in the bone marrow was reported to possess the functional plasticity to differentiate into tissue-resident or inflammatory macrophages 32, and early B cell precursors that express CD115 in fetal liver were identified 56. Our study agrees with these findings, demonstrating that the early B cell precursors can transdifferentiate to osteoclasts and that it can be mediated *via* the differentiation of the B cells to macrophages, which then differentiate into osteoclasts. The observations that monocytes/macrophages are the main precursors of osteoclasts 20, and that EPO has been shown to directly enhance osteoclastogenesis *via* EPO-R on macrophages 10 may explain our current findings concerning the differentiation of early B cell progenitors to osteoclasts and the effect of EPO in this context. We postulate that our success in identifying EYFP labeled osteoclasts in the MB1-Cre;R26R-EYFP mice (B cell lineage tracing) is due to the significantly more efficient labeling of early B cell progenitors in the MB1 lineage tracing model (Hobeika et al 47 and confirmed by our data not shown here), in contrast to a previous study, which failed to detect labeled osteoclasts 48 with CD19 lineage tracing.

An interesting question for future investigations would be whether the Pro-B cells can transdifferentiate to osteoclasts in the absence of monocytes, and whether this process is affected by inflammatory conditions which are known to stimulate osteoclastogenesis 57, 58. In our primary cultures, we could trace the differentiation of B cells to osteoclasts, but we cannot completely rule out the possibility that sporadic monocytes that did not reach the level of detection by flow cytometry were still present. This conjecture might be of relevance since there is evidence that osteoclast fusion is initiated by a subset of precursors, termed "fusion founders", capable of fusing with “fusion followers” that are unable to initiate fusion by themselves 59. The question of whether B-cell-derived preosteoclasts act as fusion founders or only as followers remains to be elucidated.

It should be noted that in culture conditions it took longer to obtain mature osteoclasts from B cells than from non-B/CD115^+^ cells (at least 8 days as opposed to about 5 days), which argues against impurities in the sorting procedure that may bias the *in vitro* experimental results. These data coupled with the small number of B cells transdifferentiating to osteoclasts *in vivo*, raise the possibility that in certain pathological scenarios characterized by bone loss 60, an increase in the population of B cell lymphocytes might contribute to bone loss as a consequence of B cell transdifferentiation into osteoclasts. In this context, ovariectomy 44 and treatment with anti-CD20 61 are two distinct medical conditions that are both characterized by an increased numbers of BM B cell precursors 62, 63 and are also associated with systemic bone loss 61, 64. Our findings prompt the question of whether B cell transdifferentiation to osteoclasts contributes to the increased bone loss in these conditions as well.

Collectively, our data advocate for a role of EPO and EPO-R signaling in bone homeostasis regulated by BM B cells. EPO may increase osteoclastogenesis in a paracrine fashion, i.e. by an upregulation of osteoclastogenic signals, as well as by direct differentiation into bone-resorbing osteoclasts. Since EPO is an effective anti-anemic therapeutic agent widely used in the clinic, our findings in mice may also be relevant in human patients.

## Supplementary Material

Supplementary figures.

## Abbreviations

BM: Bone marrow
cKD: Conditional knockdown
EPO: Erythropoietin
EPO-R: EPO receptor
M-CSF: macrophage colony stimulating factor
OPG: Osteoprotegerin
RANK: Receptor activator for nuclear factor kappa B
RANKL: Receptor activator of nuclear factor kappa-Β ligand
Tb.Th: Trabecular thickness
TRAP: Tartrate-resistant acid phosphatase
vBMD: Volumetric bone mineral density
