# Supplementary material for: Erythropoietin receptor in B cells plays a role in bone remodeling in mice
Source: Theranostics. 2020 Jul 9;10(19):8744–56. doi: 10.7150/thno.45845 (PMC7392011; doi:10.7150/thno.45845)
Supplement: Supplementary file 1 — Supplementary figures. [file thnov10p8744s1.pdf]

Figure S1

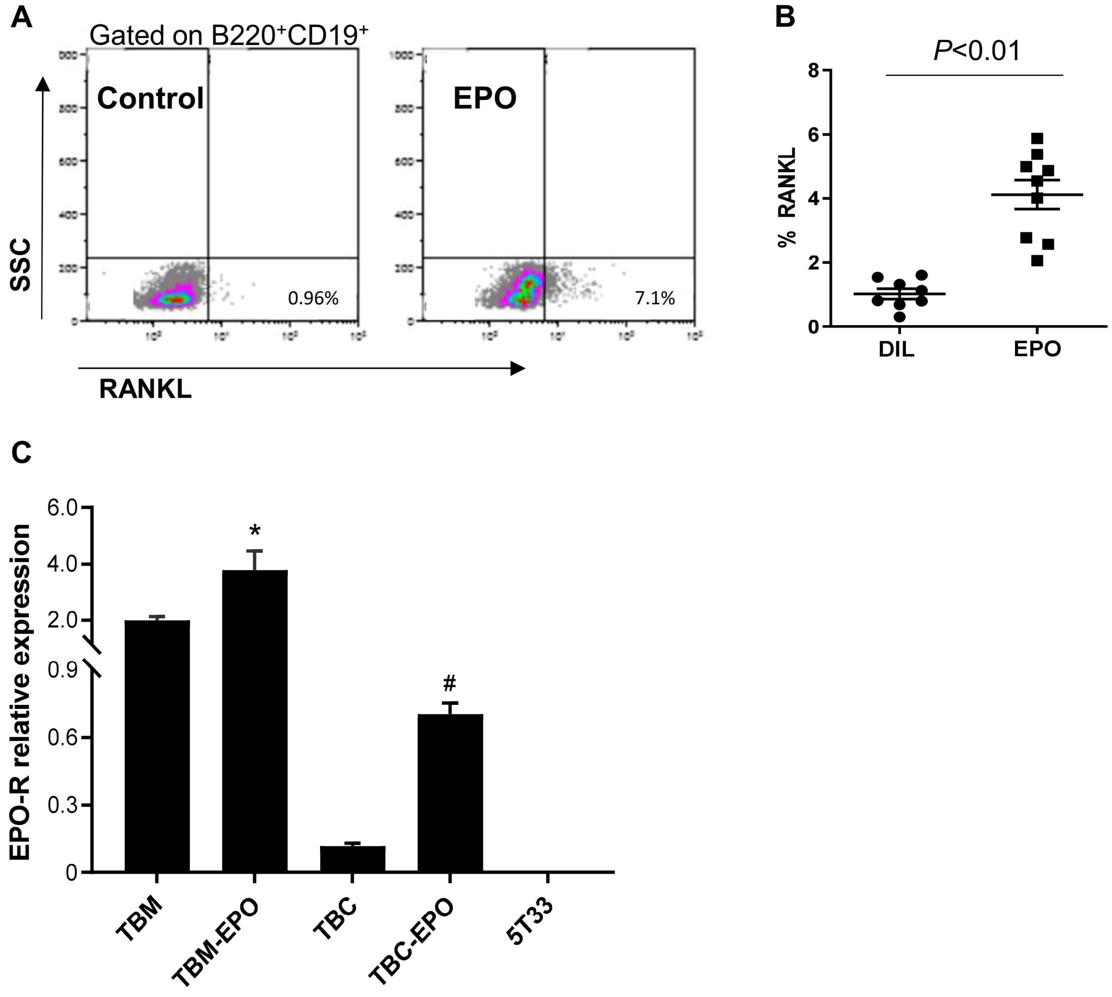

<u>Supplemental Figure 1:</u> ***The in vivo effect of EPO on the expression of membrane-bound RANKL by bone marrow B cells.*** **(A)** Representative flow cytometry density plots demonstrating the expression of surface RANKL in bone marrow (BM) B cells ($B220^+CD19^+$ cells from diluent- or EPO-injected† female mice). Percent gated $RANKL^+B220^+CD19^+$ cells are indicated. **(B)** Percent RANKL-expressing BM B cells (out of total $B220^+CD19^+$ cells). Graphs are mean ± SEM, n = 8-9 mice in each group. **(C)** EPO-R expression, as measured by RT-qPCR, in total BM (TBM) *versus* sorted total BM B cells (TBC, $B220^+CD19^+$) from either control diluent (TBM/TBC), or EPO-treated mice (TBM-EPO/TBC-EPO). Data are Mean ± SEM, > 6 mice in each group, *p* values were calculated by Student's *t*-test. * $p < 0.05$ *versus* TBM Control, #p< 0.05 *versus* $B220^+CD19^+$ control.

† EPO was administered for one week, as 3 injections of 180 U, administered every other day.

Figure S2

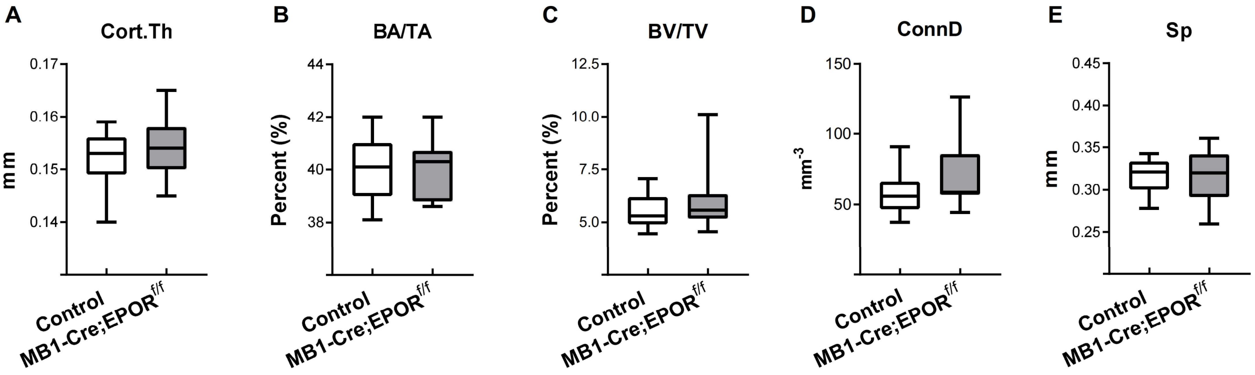

<u>Supplemental Figure 2:</u> ***Additional µCT data of female mice carrying a conditional knockdown of EPO-R in the B cell lineage (MB1-Cre;EPO-R$^{fl/fl}$)***

Cortical bone: **(A)** Cortical thickness (Cort.Th) and **(B)** Bone area per total area (BA/TA). Trabecular bone: **(C)** trabecular bone volume (BV/TV); **(D)** Connectivity density (ConnD) and **(E)** Trabecular separation (Sp). Error bars represent 5-95 percentile range. n = 11 in each group. MB1-Cre;EPO-R$^{wt/wt}$ were used as controls.

Figure S3

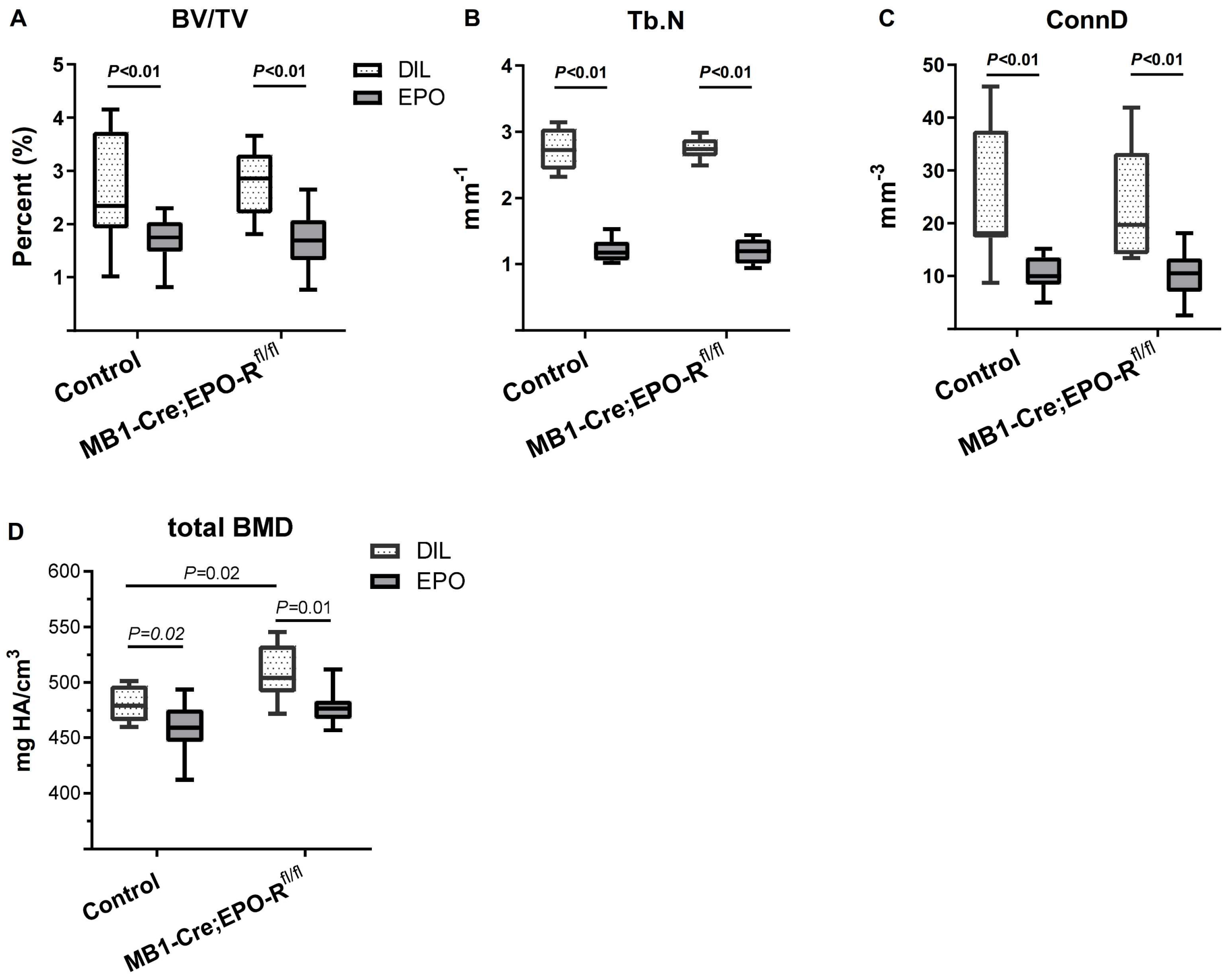

Supplemental Figure 3: ***Additional µCT data describing the effect of EPO treatment on bone mass in MB1-Cre;EPO-R$^{fl/fl}$ female mice as compared to genotypic controls***

(A) Trabecular bone volume (BV/TV); **(B)** Trabecular number (Tb.N), **(C)** Connectivity density (Conn.D) and (**D**) total bone mineral density (BMD), as assessed by µCT, in either EPO- or diluent (DIL)-treated MB1-Cre;EPO-R$^{fl/fl}$ *versus* control mice. Error bars represent 5-95 percentile. n = 9-10 in each group. MB1-Cre;EPO-R$^{wt/wt}$ were used as genotypic controls.
